# Effects of Caffeine on Voluntary Force Estimation During Isometric Exercises

**DOI:** 10.3390/sports14030090

**Published:** 2026-03-02

**Authors:** Ester Jiménez-Ormeño, Verónica Giráldez-Costas, Beatriz Lara-López, María Menchén-Rubio, Carlos Ruiz-Moreno

**Affiliations:** 1 GRUPO GIDECS, Exercise Physiology Laboratory, Faculty of Health Sciences-HM Hospitals, University Camilo José Cela, Villanueva de la Cañada, 49, 28692 Madrid, Spain; ester.jimenez@uam.es (E.J.-O.); vgiraldez@ucjc.edu (V.G.-C.); blara@ucjc.edu (B.L.-L.); mmenchen@ucjc.edu (M.M.-R.); 2 Strength Training and Neuromuscular Performance Research Group (STreNgthP_RG), Faculty of Health Sciences-HM Hospitals, University Camilo José Cela, C/Castillo de Alarcón, 49, Villanueva de la Cañada, 28692 Madrid, Spain; 3 Faculty of Health Sciences-HM Hospitals, University Camilo José Cela, C/Castillo de Alarcón, 49, Villanueva de la Cañada, 28692 Madrid, Spain; 4 Department of Physical Education, Sport and Human Movement, Universidad Autónoma de Madrid, C/Francisco Tomás y Valiente, 3, Fuencarral-El Pardo, 28049 Madrid, Spain

**Keywords:** stimulant, perception, isometric force, excitability, caffeine

## Abstract

**Background:** Caffeine is widely used as an ergogenic aid to enhance strength performance; however, its effects on perceptual accuracy during submaximal force regulation remain unclear, particularly in multi-joint isometric tasks. This study examined whether caffeine ingestion influences maximal isometric force production and the accuracy of voluntary submaximal force estimation during complex isometric exercises. **Methods:** Seventeen recreationally trained participants completed a randomized, double-blind, placebo-controlled crossover study. Participants ingested either caffeine (4 mg·kg^−1^ body mass) or a placebo before performing an isometric squat test (ISqT) and an isometric mid-thigh pull test (IMTP). Maximal voluntary contractions were assessed, followed by freely estimated submaximal efforts at 50% and 75% of perceived maximal force. Relative peak force and discrepancies between prescribed and exerted force (estimation error) were analyzed, with discrepancies calculated as the difference between exerted force and the prescribed target intensity. **Results:** Caffeine ingestion did not significantly affect relative peak force during maximal isometric efforts nor improve the accuracy of voluntary submaximal force estimation. Regardless of supplementation conditions, participants consistently misestimated submaximal efforts, tending to overproduce force, particularly at lower intensities. The IMTP showed a closer approximation to prescribed submaximal targets than the ISqT. **Conclusions:** Ingesting 4 mg·kg^−1^ of caffeine does not enhance maximal isometric force output or perceptual accuracy during voluntary submaximal force regulation in multi-joint isometric tasks. Prescribing isometric intensity based solely on perceived effort may therefore be unreliable under these specific testing conditions, particularly at lower intensities.

## 1. Introduction

The effectiveness of isometric training is linked to myriad dynamic manifestations of strength. Isometric contractions, where the muscle–tendon unit remains at a constant length, are a highly reliable means of assessing and tracking changes in force production [1].

Although isometric training involves exerting sustained force at a specific angle, depending on the approach used, it is associated with benefits such as reducing pain in affected joints and improving force production in tendons [2,3]. In addition, compared to dynamic exercises, isometric training places a lower energy demand than dynamic training, which would lead to fewer risks for the joints [4,5,6].

Isometric training, encompassing both maximal and submaximal efforts, may foster an approach towards maximal movement capacity owing to its notable mechanical transferability to dynamic exercises across diverse sporting disciplines [6,7,8,9]. Exercises such as the isometric squat (ISqT) or the isometric mid-thigh pull (IMTP) prove efficacious for enhancing execution and optimizing performance in dynamic exercises or their derivatives [2,10,11,12]. Moreover, both exercises are demonstrated to be time-effective, reliable, and associated with a low injury risk [11,13].

A strength training monitoring strategy incorporates the utilization of perceived exertion at a designated intensity level. While some studies have assessed monitoring strategies for strength training centered on perception, the predominant focus lies on dynamic strength exercises [14,15,16]. Interestingly, in dynamic strength exercises, where perception of exerted force was evaluated relative to the one-repetition maximum (1RM), perceived exertion adjusted more closely when loads approached the 1RM. However, it was concluded that this method is not an effective strategy for determining the 1RM [16]. To our knowledge, there is limited information available regarding isometric strength training and its perception of exerted force. While this methodology is prevalent in warm-up protocols, training, and rehabilitation, primarily targeting local muscle groups, its application in multi-joint movements remains largely unexplored [17,18,19].

Although frequently used interchangeably, perceived exertion and force estimation represent distinct constructs. Voluntary force estimation refers to the ability to match an intended relative force output without external feedback, whereas perceived exertion reflects the internal psychophysiological experience of effort, commonly assessed using validated scales such as the Borg RPE or CR-10 [20]. In contrast, force estimation involves the intentional regulation of motor output to achieve a target intensity, typically expressed as a percentage of maximal effort, in the absence of external feedback [15]. Accordingly, references to perceived exerted force in the present study should be interpreted as reflecting motor output regulation rather than subjective exertional sensation.

A potential confounding factor influencing force estimation tasks is caffeine intake, given its widespread use among both recreational and competitive athletes. Many individuals utilize caffeine as a strategy to augment athletic performance due to its capacity to stimulate the central nervous system [21,22,23,24,25]. The effects of caffeine on psychophysical variables include attenuation of fatigue, increased arousal, and enhanced vigilance and concentration [26,27,28]. These effects are primarily attributed to the antagonism of adenosine receptors in the brain, particularly the A_1_ and A_2A_ isoforms, resulting in heightened excitatory neurotransmission [29,30].

Studies examining the effects of caffeine on isometric training and ergogenicity are plentiful [24,25,31,32]; however, previous studies have primarily examined perceptual responses to caffeine during sustained submaximal isometric contractions or fatigue-based protocols, whereas its influence on voluntary force estimation during brief, multi-joint isometric efforts remains unclear. Interestingly, the initial studies with humans evaluated submaximal isometric response with caffeine supplementation, reporting that caffeine reduced the perceived exerted force during the initial seconds of submaximal isometric contraction [33]. The same authors, in another subsequent research, demonstrated that during 100 s of an isometric contraction of the quadriceps (50% maximal voluntary contraction), caffeine ingestion reduced force sensation during the first 10 ± 20 s of the contraction, suggesting neural effects [34]. However, all these studies were evaluated in isometric contractions until fatigue. Another possible explanation for the performance enhancement could be the substance’s ability to increase excitability through the release of neurotransmitters such as adrenaline and noradrenaline, which may lead to greater vigor in performing motor efforts [35,36,37]. Furthermore, the motivation behind these studies was to analyze the physiological mechanism in nerve firing during an isometric contraction. However, although perceptually guided isometric force production has been previously examined, existing studies have not investigated multi-joint isometric tasks or the influence of caffeine on voluntary force estimation.

Therefore, the main aim of this study was to examine whether the ingestion of 4 mg·kg^−1^ of caffeine influences force output and the accuracy of voluntary submaximal force estimation during two commonly used isometric strength tests: the isometric squat (ISqT) or isometric mid-thigh pull (IMTP). Specifically, we investigated whether caffeine alters the ability to match intended submaximal force levels (50% and 75% of perceived maximum) in the absence of external feedback, as well as maximal peak force production. Given caffeine’s known psychostimulant effects, its potential influence on voluntary force regulation was explored, while no a priori assumptions were made regarding its effects on maximal force output. As a secondary objective, the study aimed to examine whether this caffeine dose was associated with the appearance of adverse effects. Based on the available literature, it was hypothesized that caffeine ingestion would not meaningfully enhance maximal isometric force output during multi-joint tasks. Conversely, given its psychostimulant effects, caffeine was hypothesized to influence voluntary submaximal force estimation, potentially altering the accuracy with which participants matched intended force levels.

## 2. Materials and Methods

### 2.1. Participants

Seventeen participants (10 men and 7 women) were recruited for the study (age: 26 ± 6 years, body mass: 81 ± 9 kg, height: 171 ± 10 cm). Inclusion criteria for participants to be involved in the research were: (a) regular engagement in strength training in their workout area, (b) ages between 18 and 35 years, (c) absence of musculoskeletal injuries in the last 3 months, and (d) habitual caffeine consumption, assessed using a pre-study questionnaire. Participants were classified as low caffeine consumers according to the criteria described by Filip et al. [38], reporting an intake of approximately one to two cups of coffee per day. In addition, all participants completed a seven-day caffeine washout period prior to each experimental session. Exclusion criteria were: (a) smokers, (b) caffeine allergy, and (c) lack of regular physical activity (<4 days of strength training). No previous studies have examined the effect of caffeine on the accuracy of voluntary self-estimation of submaximal force during multi-joint isometric tests (ISqT and IMTP). Therefore, and in line with recommendations for transparent sample size justification, we conducted a sensitivity power analysis to quantify the smallest effect that our sample could reliably detect. A sensitivity power analysis was conducted to determine the detectable effect size given the available sample size, providing approximately 80% power to detect moderate-to-large standardized effects (d_z ≥ 0.72). This approach was adopted given the exploratory nature of the study and the known variability associated with voluntary force estimation during isometric tasks, using G*Power (Version 3.1.9.7; Heinrich-Heine-Universität Düsseldorf, Düsseldorf, Germany). The achieved sample size is comparable to prior placebo-controlled crossover studies assessing isometric performance outcomes such as the IMTP (e.g., *n* = 22–29). Given that meta-analytic estimates suggest that caffeine’s effects on maximal strength outcomes are typically small on average (≈0.20), effect sizes and 95% confidence intervals were emphasized to aid interpretation, and small effects cannot be ruled out. Before conducting the study, participants were informed about the research protocol and potential adverse effects associated with caffeine, and they signed a written informed consent form. The research protocol complied with the principles of the Declaration of Helsinki of the World Medical Association, and it was approved by the Ethics Review Board of the Universidad Camilo José Cela (code: 09_23_RFDCAF).

### 2.2. Experimental Design

A double-blind, placebo-controlled, randomized, crossover experimental design was used. The order of caffeine and placebo conditions was fully randomized across participants, and a sufficient washout period was implemented to minimize potential carryover effects. Given the acute nature of caffeine’s effects and the ≥48 h washout period implemented between sessions, residual carryover effects were considered unlikely. However, period or sequence effects were not explicitly modeled in the statistical analysis and therefore cannot be entirely excluded. Each participant visited the laboratory three times with a 48 h rest between days. The first day involved familiarization with the isometric protocols, which they had to replicate on two subsequent days within the same week to avoid training effects. On second and third day, participants ingested an opaque capsule containing 4 mg per kg of body mass of caffeine or a capsule of placebo (details provided in Section 2.4), with 150 mL of water. All experimental protocols were replicated on the days they visited the laboratory. The allocation sequence (caffeine/placebo) was computer-generated using permuted blocks of variable size by an independent investigator and managed in a centralized online randomization system (Randomizer, https://www.randomizer.org; accessed on 15 May 2023). Given that the caffeine dose was nominative (mg·kg^−1^ body mass), capsules were prepared individually by a team member not involved in data collection or analysis, using opaque, indistinguishable capsules (caffeine vs. placebo) and participant-specific kits labeled only with the participant code and period number; the code–condition correspondence remained concealed until the database was locked. To minimize carryover effects, treatment periods were separated by ≥48 h, an interval that comfortably exceeds ≥5 half-lives of caffeine in adults (mean half-life ≈ 4 h, range 2–8 h), consistent with methodological recommendations to use a washout of at least five half-lives in crossover designs.

After 45 min of capsule ingestion [39], participants performed a standard 5 min warm-up on a cycle ergometer at 50 watts, followed by 10 repetitions of air squats and joint mobility exercises. Subsequently, they underwent two isometric neuromuscular performance tests, always in the same order: (i) the isometric squat (ISqT) test and (ii) the isometric mid-thigh pull (IMTP) test. The experimental protocol in both isometric tests consisted of three series, with 2 repetitions per series (6 repetitions in total per isometric test). The first series was performed at 50% of maximum perceived effort, the second series at 75% of maximum perceived effort, and the last two repetitions at 100% of maximum effort. The rest time between the first four repetitions was 1 min, while between the fifth and sixth repetition, it was minimum at 90 s. Counterbalancing intensities was not implemented because performing maximal isometric efforts prior to submaximal trials can induce neuromuscular fatigue, altered afferent feedback, and changes in central motor drive [40], shortening performance duration in submaximal tasks following prior maximal fatiguing efforts [41]. Moreover, the fixed ascending order was selected to ensure participant safety during maximal multi-joint isometric tests and the standardization of testing procedures [17]. After completion of all experimental sessions, participants were asked to retrospectively indicate which condition they believed they had received. This question was included to describe participants’ subjective perceptions and beliefs rather than to assess blinding during task execution. Importantly, participants were unaware of the experimental condition during all testing sessions.

Twenty-four hours post exercise, participants completed a questionnaire assessing side effects commonly associated with caffeine consumption. The questionnaire utilized a 1–10-point scale for each item and has been previously validated in athletes to quantify the magnitude of side effects attributed to caffeine supplementation [42].

### 2.3. Isometric Testing Protocols

Technical instructions were provided for each subject before each test to perform a correct execution. The ISqT test was assessed at a relative knee angle of 90° because this angle reflects the sticking point during the squat exercise [43,44]. The relative knee angle was measured using a handheld goniometer (Jamar^®^, Patterson Medical, Warrenville, IL, USA) by the lead researcher. Participants assumed a squat position on the force plate with their feet shoulder width apart, maintaining a near-vertical trunk orientation, while the immoveable bar remained positioned above the posterior deltoids to ensure neutral pelvic and spinal alignment during each effort, mitigate injury risk and allow for effective transfer of force. Prior to each trial, this stance was confirmed, and joint angles were verified. Participants’ stance widths and height bar were recorded to ensure consistency between trials [43,45].

The IMTP test was required to maintain an upright trunk (≤10° forward lean), with the optimal knee (125–145°) and hip (140–150°) angles, shoulder girdles retracted and depressed above or slightly behind the vertical plane of the bar, feet hip-width apart, knees underneath and in front of the bar, and thighs in contact with the bar [17]. The angles were measured using a handheld goniometer (Jamar^®^, Patterson Medical, Warrenville, IL, USA). by the lead researcher. Individual’s grip width, foot position and bar height were recorded to standardize across sessions [46]. During this test, participants used lifting straps to prevent grip strength being a limiting factor [17].

The vertical ground reaction force (vGRF) applied to the whole-body center of mass during each isometric test was recorded using a wireless dual-force plate system with a sample rate of 1000 Hz (Hawkin Dynamics Inc., Westbrook, ME, USA). The validity of Hawkin Dynamics hardware and software has been demonstrated in previous studies [47,48,49]. The ISqT and the IMTP test were performed with a portable isometric pull rack (Absolute Performance, Inc., Broomfield, CO, USA). The force plates were placed on this rack on flat, level ground and zeroed before each repetition was recorded. The rack had two vertical elements with holes to insert a metal bar, which was fixed at the height of the participants’ position.

Participants held the position in each test to obtain a steady weighing period for 1 s prior to each isometric effort. Although the duration of individual contractions was not strictly fixed at a single time point (approximately 5 s), consistency in execution was ensured through standardized verbal instructions and real-time monitoring of force output to identify force plateaus and peak values [17]. For each trial, participants were verbally instructed to have minimal pre-tension and push as hard (50% of maximum perceived effort, 75% of maximum perceived effort or 100% of maximum effort) and as fast as possible [17]. For each trial, participants were verbally instructed to have minimal pre-tension and push as hard (50% of maximum perceived effort, 75% of maximum perceived effort or 100% of maximum effort) and as fast as possible [50], pushing the ground away while maintaining body posture to ensure force application [17]. Efforts commenced after an audible beep provided by Hawkin Dynamics software (https://www.hawkindynamics.com/hawkin-dynamics-software, accessed on 23 February 2026). In real time, the researchers observed the force–time traces, and trials were finished when a plateau in the trace was visually observed for a period of 1–2 s, indicating that peak force had been achieved [44]. Participants performed an additional trial if they lost their posture, had a coefficient of variation >15% between trials based on peak force, or did a countermovement before the start of the pull [17]. Moreover, during the maximal trials, in both ISqT and IMTP tests, motivational techniques were provided so that the participants would perform isometric strength at their maximum effort [17]. This protocol was repeated under all conditions, regardless of the substance ingested.

### 2.4. Standarizations

One week prior to the protocol, participants visited the laboratory to be weighed without clothing (±50 g, Radwag, Radom, Poland). Participants were instructed to abstain from consuming any foods, products, or substances containing caffeine or any other stimulants or ergogenic aids one week before the experimental test. This restriction was to be maintained throughout the entire experimental protocol. They were also advised to continue consuming their usual macronutrient balance in their diet and abstain from engaging in strength training throughout the study.

The experimental protocol was conducted over one week, during three sessions (Monday, Wednesday and Friday), which were synchronized to the identical hour as the first session (familiarization) to accommodate circadian rhythmicity [51].

On experimental days (second and third sessions), participants consumed an opaque capsule containing 4 mg of caffeine (HSN, Granada, Spain) per kg of body mass or a placebo (cellulose; Guinama, Valencia, Spain). The experimental tests were performance 45 min after the ingestion of caffeine or the corresponding placebo. Between each experimental session, a minimum of 48 h elapsed to allow for substance clearance from the organism and to facilitate neuromuscular recovery [39].

### 2.5. Statistical Analysis

The data are presented as the mean and standard deviation. Additionally, 95% confidence intervals (CIs) were calculated for the exerted force, expressed as a percentage of maximal effort during submaximal trials (50% and 75%) to provide an estimate of the precision of voluntary force estimation. Descriptive statistics were calculated for each condition and effort. Normality distribution of the variables was conducted using the Shapiro–Wilk test with the Jamovi statistical software package (version 2.3.18, Sydney, Australia). A repeated measures analysis of variance (ANOVA) was employed to examine the main effect of caffeine, the main effect of effort (50%, 75% or 100%), and the interaction between both factors (caffeine × effort). The Tukey post hoc test was applied for pairwise comparisons at different intensities of the condition. Sphericity was assessed using Mauchly’s test, and when violations were detected, Greenhouse–Geisser corrections were applied.

The percentages of actual exerted force during 50% of maximum perceived effort and 75% of maximum perceived effort were calculated according to 100% of maximum effort, in both conditions (caffeine and placebo), based on relative peak force, defined as the peak instantaneous vertical ground reaction force applied during the isometric test, normalized for body mass. To analyze disparities between the obtained percentages (exerted force and perceived exerted force), Student’s *t*-test for related samples was used for variables with a normal distribution, and the Wilcoxon test was used for those with a non-normal distribution. These statistical tests were also applied to determine possible differences in adverse effects between the two conditions (caffeine and placebo). Statistical significance was set to *p* ≤ 0.05.

## 3. Results

**Figure 1:**
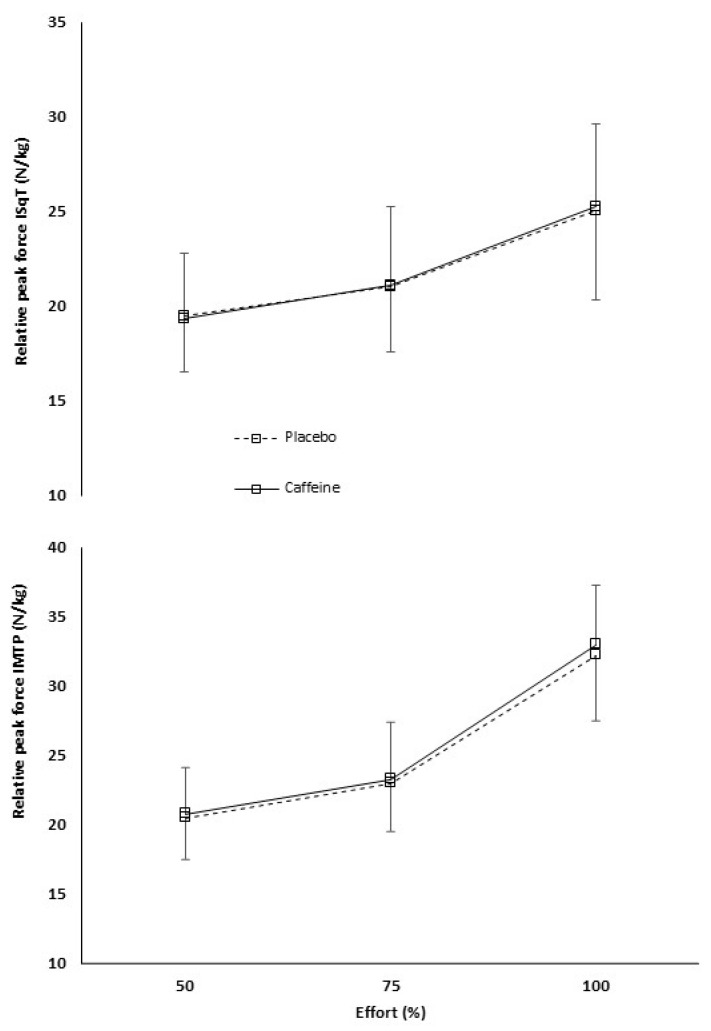
Relative peak force (N/kg) during efforts of 50%, 75% and 100% with caffeine or placebo. ISqT: isometric squat test; IMTP: isometric mid-thigh pull test.

**Figure 2:**
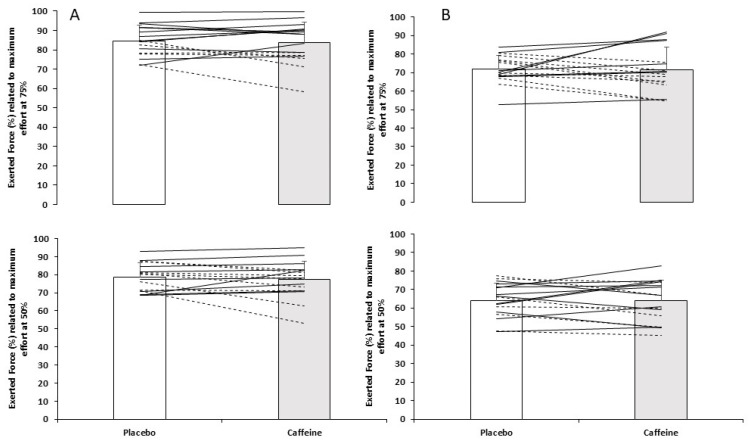
Relative peak force exerted at 50% and 75% of maximum perceived effort related to maximum effort with caffeine or placebo. Column (**A**) depicts isometric squat test and column (**B**) depicts isometric mid-thigh pull test. Lines represent individual responses. Solid lines indicate participants who improved from placebo to caffeine, whereas dashed lines indicate participants who showed reduced performance under caffeine compared to placebo.

During the ISqT test, caffeine did not have a main effect on relative peak force (F = 0.071, *p* = 0.93). In pairwise comparisons by effort, there were no significant differences in relative peak force when participants ingested caffeine or placebo during a submaximal effort of 50% (placebo: 19.5 ± 3.0 N/kg, caffeine: 19.4 ± 3.4 N/kg; *p* = 0.79), a submaximal effort of 75% (placebo: 21.0 ± 3.5 N/kg, caffeine: 21.1 ± 4.1 N/kg; *p* = 0.82), or the maximal effort of 100% (placebo: 25.0 ± 4.7 N/kg, caffeine: 25.3 ± 4.4 N/kg; *p* = 0.49) (Figure 1, upper panel). In relation to the relative peak force obtained in the maximum repetition, the participants overestimated their efforts, expressing 77.7 ± 7.8% in the placebo condition and 77.2 ± 10.1% in the caffeine condition when they were asked for 50% of maximum perceived effort (*p* = 0.42), and 84.6 ± 8% with placebo and 83.8 ± 10.4% with caffeine when they were asked for a 75% of maximum perceived effort (*p* = 0.63) (Table 1 and Figure 2A).

During the IMTP test, there was no main effect of the substance on relative peak force (F = 0.262, *p* = 0.77). Regarding the comparison by effort, either there were no significant differences in submaximal efforts of 50% (placebo: 20.4 ± 3.3 N/kg, caffeine: 20.7 ± 4.0 N/kg; *p* = 0.59) or 75% (placebo: 23.0 ± 3.6 N/kg, caffeine: 23.3 ± 4.5 N/kg; *p* = 0.65), nor during the maximal effort of 100% (placebo: 32.2 ± 5.2 N/kg, caffeine: 32.9 ± 6.6 N/kg; *p* = 0.35) (Figure 1, lower panel). Participants overestimated peak force during the 50% effort compared to 100% (placebo: 62.7 ± 12%, caffeine: 63.9 ± 11.2%; *p* = 0.88), and it was underestimated during the 75% effort (placebo: 70.2 ± 9.5%, caffeine: 71.5 ± 12.1; *p* = 0.89) (Table 1 and Figure 2B).

Regarding individual responses, participants were asked, after completion of the entire experimental protocol, to indicate which condition they believed corresponded to caffeine. In total, 75% reported correct identification; importantly, this assessment was conducted only after all testing sessions had concluded and did not occur during task execution. During 50% and 75% of maximum perceived effort in the ISqT test, 50% of the participants increased peak force when they ingested caffeine, and during the maximum effort, 37.5% of the participants’ results increased when supplemented. During the IMTP test, 37.5% of the participants increased intensity when they consumed caffeine at the estimated percentages of 50% and 75% of maximum perceived effort, respectively. As an individual response during the maximum effort, 62.5% increased intensity when they ingested caffeine (Figure 2).

Regarding adverse effects reported the next day, the caffeine condition showed a significant increase in vigor and diuresis (Table 2).

## 4. Discussion

The aim of this study was to analyze whether the ingestion of 4 mg·kg^−1^ body mass of caffeine affects relative peak force and the accuracy of self-estimated submaximal intensities (50%, 75%) during two isometric tests: the ISqT and IMTP. The present findings indicate that caffeine ingestion neither enhances maximal isometric force output nor improves the accuracy of voluntary submaximal force estimation during multi-joint isometric tasks. Importantly, regardless of the supplementation condition, participants systematically misestimated the prescribed force levels, tending to overproduce force relative to the intended targets, particularly at lower intensities. This suggests that caffeine-induced increases in arousal do not translate into improved perceptual–motor calibration during complex isometric tasks performed without external feedback.

No significant differences in relative peak force were observed between caffeine and placebo conditions at any effort. Although small increases in peak force were observed following caffeine ingestion in both tests (1.3% in ISqT and 2.1% in IMTP), these changes were small and of limited practical relevance. These findings are consistent with previous studies reporting minimal or inconsistent ergogenic effects of caffeine on maximal isometric performance in complex tasks [24,52,53].

Multi-joint isometric tasks such as the ISqT and IMTP impose substantial demands on intermuscular coordination, postural control, and joint-angle specificity, which may limit the translation of increased central nervous system excitability into measurable gains in force output [13,17]. In such contexts, despite increased arousal, caffeine’s stimulatory effects may not be sufficient to overcome the integrative neuromuscular demands required for precise and maximal performance. This limitation is particularly relevant in isometric strength assessments, where effort regulation and joint-angle specificity constrain the manifestation of acute ergogenic adaptations [35].

To the authors’ knowledge, this is the first study to evaluate the accuracy of voluntary force estimation across multiple submaximal intensities during multi-joint isometric strength tests following caffeine ingestion. While improvements in maximal isometric force have previously been reported in single-joint or localized muscle actions [19,24], evidence from complex, multi-joint tasks remain equivocal. It should be acknowledged that training status may influence caffeine responsiveness, as well-trained individuals typically demonstrate greater neuromuscular efficiency, enhanced motor-unit recruitment capacity, and more refined perceptual calibration. These factors may modulate the magnitude or consistency of caffeine’s ergogenic effects. Therefore, differences in participant training background may partially explain similarities or discrepancies between the present findings and previous IMTP studies conducted in well-trained populations [54,55,56].

Participants consistently overestimated the prescribed submaximal intensities, particularly during the ISqT. In this test, exerted force exceeded the intended target by 27.2% at the 50% level and by 8.8% at the 75% level. In the IMTP, overestimation was 13.9% at 50%, while force output at 75% was closer to the expected target (−3.5%). These findings indicate a systematic bias in voluntary force estimation during multi-joint isometric actions, particularly at lower prescribed intensities, in recreationally trained individuals. In the context of the present study, accuracy refers to the degree of agreement between the prescribed target intensity and the actual force produced, operationalized as estimation error relative to maximal voluntary contraction. Accordingly, positive deviations reflect systematic overestimation of the intended effort, whereas values closer to the prescribed target indicate more accurate voluntary force regulation.

Caffeine ingestion did not reduce the discrepancy between perceived and exerted force. Despite the absence of ergogenic effects on submaximal force output, participants reported a greater sensation of vigor following caffeine intake, consistent with previous reports on caffeine-related side effects [42]. This finding aligns with the well-documented psychostimulant effects of caffeine on the central nervous system, including increased alertness, arousal, and perceived readiness [26,27]. However, heightened arousal does not necessarily translate into improved perceptual motor calibration. In the absence of external feedback, increased central activation may enhance readiness without improving and potentially impairing the accuracy of voluntary force regulation, as previously suggested in precision-based motor tasks [37].

Collectively, these findings indicate that caffeine-induced arousal does not improve the accuracy of voluntary force regulation during tasks requiring matching between perceived effort and force output, particularly in complex multi-joint isometric actions performed without external feedback. Importantly, the present results highlight a consistent lack of accuracy when submaximal efforts are prescribed relative to maximal strength. Given that isometric warm-up, training, and testing protocols frequently include repetitions performed at a percentage of maximal force [17,43,45], this imprecision may have important implications for the standardization, validity, and interpretation of isometric strength assessments.

In this sense, the present results align with those reported byWest et al. [19], who also observed an overproduction of force or perceptual underestimation during submaximal isometric actions. This phenomenon may be theoretically explained by the reduced sensitivity of large muscle groups to accurately discriminate lower force levels, as suggested in the previous literature, such as the knee extensors involved in the ISqT and IMTP, to accurately discriminate and reproduce lower force levels [57]. Additionally, limited familiarity with submaximal isometric contractions and the instructions provided to participants may further contribute to this systematic error [13]. Notably, previous research has shown that standard warm-up or familiarization procedures are often insufficient to ensure accurate calibration of perceived effort relative to maximal strength, even in resistance-trained individuals [43,45].

Several limitations of the present study should be acknowledged. First, a limitation of the present study is the fixed order of effort intensities, with submaximal trials always preceding maximal efforts. Although participants completed a familiarization session, this fixed progression may have influenced perceptual calibration and neuromuscular readiness within sessions. However, this approach was selected to ensure participant safety and protocol standardization, is consistent with commonly used protocols in isometric testing, and was applied identically across conditions [17,18]. Second, no pre-assessment of participants’ readiness or subjective state was conducted prior to each testing session. While previous studies have suggested that such factors may influence perceptual and performance outcomes [34,58], their omission reflects typical applied testing scenarios. Additionally, although participants were blinded to supplementation during testing, a proportion of participants retrospectively identified the caffeine condition after completion of all sessions. While this occurred after task execution and did not influence performance during testing, expectancy effects cannot be entirely ruled out and should be considered when interpreting perceptual outcomes. Finally, only a single caffeine dose was examined. Although this dose is commonly used in strength-related research [31], future studies should explore potential dose–response effects on voluntary force estimation and perceptual accuracy.

## 5. Conclusions

In summary, the ingestion of 4 mg·kg^−1^ of caffeine did not enhance maximal isometric force production nor improve the accuracy of voluntary submaximal force estimation during either the ISqT or IMTP. Regardless of the supplementation condition, participants’ force output during freely estimated submaximal isometric efforts did not correspond to the intended relative intensities, indicating a consistent mismatch between perceived and exerted force. Among the two tests, the IMTP showed the closest approximation to the prescribed submaximal targets. From a practical perspective, these findings suggest that, under the specific conditions of the present study, prescribing isometric exercise intensity based solely on perceived percentages of maximal effort may be unreliable, particularly at lower intensities. This interpretation applies to multi-joint isometric tasks performed by recreationally trained individuals without external feedback, and should be considered within the methodological context of the present study. Therefore, when this approach is used in warm-up, rehabilitation, or training contexts, higher submaximal targets, external feedback, or explicit instruction aimed at force regulation should be considered. Future research and applied protocols should also incorporate more robust familiarization strategies to improve the accuracy of self-perceived force during submaximal isometric actions.

## Figures and Tables

**Table 1:** Exerted force (%) relative to maximal effort during submaximal isometric contractions. Mean ± SD and 95% confidence intervals (CIs).

Test	Variable	Placebo	Caffeine	*p*
ISqT	Exerted force (%) related to maximum effort at 50%	77.7 ± 7.8 (95% CI: 74.6–82.7)	77.2 ± 10.1 (95% CI: 72.0–82.4)	0.42
	Exerted force (%) related to maximum effort at 75%	84.6 ± 8 (95% CI: 80.5–88.7)	83.8 ± 10.4 (95% CI: 78.5–89.2)	0.63
IMTP	Exerted force (%) related to maximum effort at 50%	64.2 ± 9.3 (95% CI: 59.4–69.0)	63.9 ± 11.2 (95% CI: 58.2–69.7)	0.88
	Exerted force (%) related to maximum effort at 75%	70.2 ± 9.5 (95% CI: 67.9–75.7)	71.5 ± 12.1 (95% CI: 65.3–77.7)	0.89

ISqT: isometric squat test; IMTP: isometric mid-thigh pull test.

**Table 2:** Adverse effects recorded after both experimental conditions.

Variable Perceptive	Placebo Mean ± SD	Caffeine Mean ± SD	*p*
Nervousness (a.u.)	1.7 ± 1.7	4.2 ± 3.6	0.06
Vigor (a.u.)	1.8 ± 1.6	5.2 ± 3.6	**0.01**
Irritability (a.u.)	1.6 ± 1.9	1.5 ± 1.1	0.82
Muscle pain (a.u.)	1.2 ± 0.6	1.0 ± 0.0	0.34
Headache (a.u.)	1.0 ± 0.0	1.2 ± 0.6	0.34
Gastrointestinal distress (a.u.)	1.0 ± 0.0	2.2 ± 2.7	0.12
Diuresis (a.u.)	1.2 ± 0.6	2.6 ± 2.6	**0.04**

(a.u) arbitrary units.

## Data Availability

The data that support the findings of this study are available from the corresponding author upon reasonable request due to the dataset containing individual-level performance and perceptual data collected in a controlled laboratory setting. Although the data are anonymized, public deposition was not initially planned, and access is therefore restricted to ensure appropriate use and interpretation. Nevertheless, the data can be shared with interested researchers upon reasonable request to the corresponding author for academic and non-commercial purposes.

## Abbreviations

The following abbreviations are used in this manuscript:

IMTP	Isometric mid-thigh pull
ISqT	Isometric squat
a.u	Arbitrary units
vGRF	Vertical ground reaction force
ANOVA	Analysis of variance
